# Patient and Physician Perspectives on Asthma and Its Therapy in Romania: Results of a Multicenter Survey

**DOI:** 10.3390/medicina57101089

**Published:** 2021-10-12

**Authors:** Dragos Bumbacea, Carmen Panaitescu, Roxana Silvia Bumbacea

**Affiliations:** 1 Department of Cardio-Thoracic Medicine, “Carol Davila” University of Medicine and Pharmacy, 020021 Bucharest, Romania; 2 Department of Pneumology and Acute Respiratory Care, Elias Emergency University Hospital, 011461 Bucharest, Romania; 3 Department of Functional Sciences, Physiology, Center of Immuno-Physiology and Biotechnologies (CIFBIOTEH), Victor Babeș University of Medicine and Pharmacy, 300041 Timisoara, Romania; cbunu@umft.ro; 4 Center for Gene and Cellular Therapies in Treatment of Cancer—OncoGen Center, Pius Brinzeu County Clinical Emergency Hospital, 300723 Timisoara, Romania; 5 Department of Allergology, “Carol Davila” University of Medicine and Pharmacy, 020021 Bucharest, Romania; roxana.bumbacea@umfcd.ro; 6 Department of Allergology, “Dr. Carol Davila” Nephrology Clinical Hospital, 010731 Bucharest, Romania

**Keywords:** asthma, asthma therapy, severe asthma, patient behaviour, patient attitudes

## Abstract

*Background and Objectives*: Patient’s behaviours, attitudes and beliefs related to asthma and its treatment were shown to influence the adherence to therapy and the level of asthma control. This survey aimed to assess the level of asthma control and patient-reported behaviours, attitudes and expectations related to their disease in Romanian patients. *Materials and Methods*: This cross-sectional quantitative survey was performed in February-March 2019 and enrolled 70 specialist physicians experienced in asthma management and 433 asthma patients under their care. *Results*: Of the 433 patients enrolled, 19.4% had mild asthma, 60.5% moderate asthma and 20.1% severe asthma. For the previous 12 months, asthma symptoms, exacerbations and emergency room visits were common in the sample analysed, with significantly higher figures in severe asthma patients (*p* < 0.001). The most important treatment goal for asthma patients was participation in all activities of daily living, while for physicians this was preventing asthma exacerbations. The valuation of the treatment goals was different between patients with severe asthma and those with mild and moderate forms. Based on the patients’ responses, 3 attitude clusters were identified: empowered savvy (36.5% of the patients), pessimistic non-compliers (43.2%), and anxious strugglers (20.3%). “Empowered savvy” had the lowest frequency of severe asthma, the highest adherence to maintenance therapy and the highest level of confidence in the effectiveness of asthma medication. The opposite of this attitude cluster is the “anxious strugglers”, containing more patients with severe asthma, a higher score for worries about asthma therapy and better self-reported knowledge of their treatment, contrasting with a proportion of 25% taking maintenance therapy only when having breathing difficulties. *Conclusion:* Asthma control in Romania remains poor, with frequent exacerbations and hospitalizations. The differences in treatment goals found between patients and physicians and between different asthma severity groups suggest the need for more patient-centred approaches.

## 1. Introduction

Asthma is one of the most common non-communicable chronic diseases, with an estimated number of 272 million persons affected worldwide in 2017 [1]. In Europe, approximately 30 million persons are living with asthma, and the total annual costs to society due to indirect costs and direct healthcare expenditures predominantly related to outpatient treatment are estimated to be around 34 billion Eur [2]. As opposed to mild and moderate forms that can be controlled with appropriate treatment, severe asthma is refractory to maximal optimized therapy and to strategies addressing contributory factors, such as inhaler technique and adherence [3]. It affects only 3–10% of patients diagnosed with asthma but has a large impact on patients’ life and is responsible for a large proportion of asthma-associated economic burden [4,5,6].

Despite the availability of effective asthma therapy, the adherence remains unsatisfactory, with rates ranging between 30% and 70% [7]. Non-adherence to therapy has been associated with poor asthma control and outcomes (including asthma death), increased healthcare resource utilization, and increased indirect costs [8,9]. Causes of non-adherence to asthma therapy are complex and a key factor seems to be related to patient behaviour, itself influenced by patient’s attitudes and beliefs related to the disease and its management [10]. Patient surveys showed that patients overestimate the level of their asthma control [11,12]. Additionally, it has been shown that even patients with persistent asthma do not believe their disease is chronic and tend to only use as-needed medication; hence the adherence increases only when the need for symptom prevention is perceived as high [13,14,15]. The over-reliance on short-acting beta-agonists (SABA) and underuse of inhaled corticosteroids (ICS) due to patients’ poor understanding of the chronic inflammatory nature of the disease were previously shown as some of the root causes of non-adherence [16]. To reverse this behaviour, the Global Initiative for Asthma (GINA) in its updated guidelines no longer recommends as-needed SABA as the only therapy for mild asthma in adults and adolescents [17].

In Romania, limited data is available on asthma prevalence and control [18,19] and no published data on patients’ attitudes related to asthma and its therapy currently exists. Given the potential to improve asthma control by understanding and addressing patient-related factors, the aim of this survey was to assess the patient-reported behaviours, attitudes and expectations related to asthma and its treatment in Romanian patients. These data would result in a better understanding of patients’ needs, providing useful insights for physicians to optimally tailor their patients’ management.

## 2. Materials and Methods

### 2.1. Survey Design and Population

The SABA Trends IN Over-reliance (SABATINO) was a cross-sectional quantitative questionnaire-based survey conducted from 1st of February 2019 to 13th of March 2019. The participants were specialist physicians involved in asthma patients’ management and patients under their care. The sample of physicians consisted of pulmonologists and allergists randomly selected from the national database. Physicians were recruited using computer-assisted telephone interviews (CATI) methodology. The eligible physicians which were interested in participating received an online invitation to the survey with a questionnaire to complete from patients’ medical charts. The following inclusion criteria applied for physicians: between 3 and 35 years since obtaining the specialist physician degree, treating at least an average number of 20 asthma patients per month and currently monitoring at least 5 asthma patients with any type of treatment except maintenance and reliever therapy (MART) in 1 inhaler regimen. Physicians unable to fill out the survey based on patients’ medical charts were excluded. To ensure national representativity, we aimed to enroll 30% of physicians from the capital of the country and the remaining from other geographical regions of Romania. The physicians which participated were asked to enroll at least 5 adult asthma patients under their current care, randomly selected over 2 weeks period, but no more than 1 patient per day. At the time of their visit to the doctor’s office, the patients selected by their treating physician to participate in SABATINO were invited to complete a paper questionnaire specifically designed for this survey. Inclusion criteria for patients were: aged 18 years or older, men or women, with physician-diagnosed asthma irrespective of their disease duration and receiving any type of maintenance treatment except the MART regimen. Eligible patients should have been prescribed SABA therapy at least once or have been previously informed about reliever therapy, irrespective if at the time of the survey they were using SABA.

As this was a market research survey, no ethics committee approval was required as per local regulations. Data collection was compliant with General Data Protection Regulation (GDPR). No information allowing patient identification was collected. The information allowing physicians’ identification was not transferred from the company performing the data collection to the sponsor of this survey. All of the patients and physicians were informed about data collection and their rights related to GDPR and provided written informed consent before data collection.

### 2.2. Data Collection

The SABATINO survey was developed by the market research organization ISRA Center (Bucharest, Romania). Questionnaires for physicians were used to collect data on medical specialty, experience (years of practice, average number of patients with asthma seen in a month) and asthma treatment goals. Patients’ data collected by physicians from medical charts were anonymized and included the following variables: disease severity at the time of diagnosis and of the present survey, presence and type of allergies, number of exacerbations and hospitalizations during the past 12 months and current treatment for asthma.

Patients’ questionnaire included 29 items grouped in the following main sections: socio-demographics, asthma diagnosis and manifestations, current asthma treatment, habits connected to and expectations from their asthma treatment, current status of the disease, including asthma-related healthcare use in the past 12 months and impact of the disease on their activities in the last month and year before survey enrollment, patients’ attitude towards asthma and sources of information about asthma. An English version of questionnaires for physicians and patients are included in the Supplementary Table S1.

### 2.3. Statistical Analysis

Responses were analysed separately for patients and physicians in the overall populations and stratified by the severity of asthma of the patients and the specialty of the physicians. Patients with severe and very severe asthma were analysed together as one group (severe asthma). Descriptive analysis of the responses was provided using mean ± standard deviation (SD) and frequency. The differences in patients’ responses between asthma severity groups were evaluated using analysis of variance (ANOVA) and chi-square methods and a *p*-value of less than 0.05 was considered significant.

To define patient typologies (clusters of attitudes) a combination of k-means and hierarchical clustering in Convergent Cluster & Ensemble Analysis System (Sawtooth Software, Provo, UT, USA) was used. The first step was to identify the efficient options from a statistical/mathematical point of view using homogeneity (respondents from each attitude cluster to be relatively similar between them on the investigated dimensions), heterogeneity (significant differences between attitude clusters, even if common pillars can exist) and segment dimension relatively balanced (for example not having 80% of the sample in one attitude cluster). Following this, the options/solutions were logically analysed to check their plausibility in the market context. Solutions with attitude clusters that had large overlaps, no coherent profile (no statements matching them) and too large groups to be compared with the others were removed.

## 3. Results

### 3.1. Socio-Demographic Characteristics

Of the 430 physicians contacted, 394 gave a positive response via phone and received further information via email. Of these, 70 fulfilled the inclusion criteria, had no exclusion criteria, and accepted to participate (15 allergists and 55 pulmonologists) and were enrolled in this survey. They had a balanced distribution of number of years of experience, geographic location and average number of asthma patients seen per month. Study physicians enrolled 433 asthma patients. Patients were mostly women, with a mean age of 49.8 years (range 18–89 years), mostly employed or retired, and with a balanced distribution of education history (Table 1).

### 3.2. Clinical Characteristics and Indicators of Asthma Symptoms Control

Of the 433 enrolled patients, 19.4% were classified as mild asthma, 60.5% as moderate asthma and 20.1% as severe asthma. The mean duration of asthma was 8.5 years, with a steady increase from mild to moderate and severe forms (*p* = 0.002). Physicians reported on average 1.8 asthma exacerbations and 1.0 hospitalizations for asthma per patient in the previous year. The average yearly number of exacerbations and hospitalizations due to asthma was higher in the more severe asthma patients (*p* < 0.001). The same trend was noticed in patient-reported outcomes. A higher percentage of severe asthma patients reported emergency room visits for asthma and overnight hospital stays during the last year (*p* < 0.001). Moreover, the number of days off-work or inability to perform usual activities during the past year was higher in severe asthma patients compared with mild-to-moderate ones (*p* < 0.001; Table 2).

### 3.3. Asthma Therapy

Overall, based on medical charts, 94.0% of patients participating in SABATINO survey had maintenance therapy with a separate reliever therapy prescribed at the time of the survey, and 6.0% had reliever therapy only. Severe asthma patients were prescribed more reliever and maintenance medication than mild-to-moderate ones, mostly biological therapy, oral steroids, anticholinergic and antileukotrienes (Table 3).

Based on the patients’ completed questionnaires, 86.1% of patients reported regular administration of their asthma medication. Reliever medication was used by 79.7% of patients for symptom relief only and 19.2% also used it to prevent asthma symptoms. Maintenance medication was used daily to prevent asthma symptoms by 81.3% of those using this type of medication, with 17.9% of the patients reporting the use of their maintenance medication only when experiencing asthma symptoms. Per severity groups, the use of maintenance therapy only when having asthma symptoms was reported by 10 patients (12.2%) with mild asthma, 45 patients (18.1%) with moderate asthma and 18 patients (23.4%) with severe asthma (*p* = 0.390).

### 3.4. Valuation of Treatment Goals

**Figure 1:**
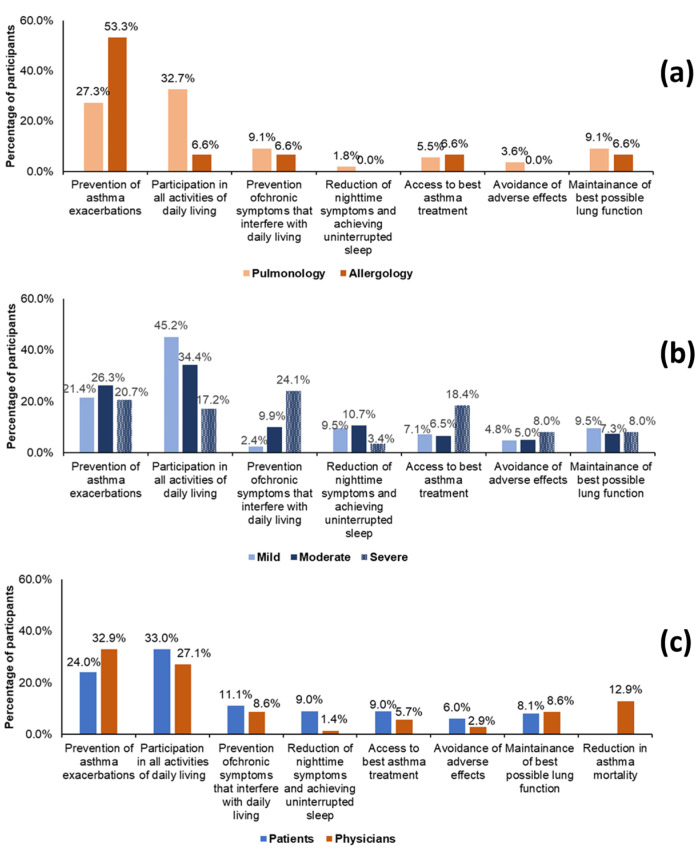
Valuation of treatment goals in physicians according to their specialty (**a**), in patients according to asthma severity (**b**) and in physicians as compared to patients (**c**). “Reduction in asthma mortality” was not a treatment goal in patient questionnaire due to cultural reasons.

The most important treatment goals frequently identified by patients were: participation in all activities of daily living, prevention of asthma exacerbations and prevention of chronic symptoms that interfere with daily living. The most important treatment goals most frequently identified by physicians were preventing asthma exacerbations, allowing the person to participate in all activities of daily living and preventing asthma mortality. Valuation was different between pulmonologists and allergists and between patients with severe asthma and those with mild and moderate forms (Figure 1).

### 3.5. Patients Attitudes toward Asthma

The patients in the survey had similar attitudes on the effectiveness of the therapy and the ease of use of any medication irrespective of their asthma severity. However, patients with severe asthma had greater concerns about their therapy and the burden of asthma medication; thus, they scored higher in questions related to worries on having breathing difficulties, the use of medication when feeling well and self-adjustment of medication (Supplementary Table S2).

Three attitude clusters were identified based on patients’ responses to questions on attitudes towards asthma and its therapy (Supplementary Table S3): empowered savvy (36.5% of the patients), pessimistic non-compliers (43.2% of patients), and anxious strugglers (20.3% of the patients).

“Empowered savvy” patients were aware and knowledgeable of their condition and felt in control even when the worsening of their symptoms occur. Compared to the other clusters, the patients in this cluster generally had a higher level of education, were less likely to be smokers and had the lowest mean number of exacerbations and hospitalizations during the previous 12 months. Furthermore, they more frequently reported administering their asthma medication, the use of reliever therapy when coughing or having breathing difficulties and daily use of maintenance therapy to prevent asthma symptoms (Table 4).

The patients in the “pessimistic non-compliers” cluster had a limited understanding of asthma therapies and as a result, they considered the therapy a burden, leading to complaints about price and multiple inhaler usage, and embarrassment related to inhaler usage in public. Lacking the knowledge or understanding about the ways to prevent a worsening of symptoms, these patients felt scared and worried, unable to manage by themselves such situations. Compared to the other clusters, patients in this attitude cluster were older, had a lower level of higher education and reported the lowest frequency of administering their asthma medication (Table 4).

The patients in the “anxious strugglers” cluster reported a good knowledge of asthma therapies and management of symptoms but exhibited worries regarding the efficiency and potential side effects of their medication. They were worried about taking too much medication when feeling well, and therefore they preferred to adjust the doses. The patients in this attitude cluster were more likely to be smokers, with severe asthma and had more exacerbations and hospitalizations during the previous 12 months. They also more frequently reported the use of reliever medication to prevent asthma symptoms and of maintenance therapy only when coughing and having breathing difficulties and less frequently daily (Table 4).

## 4. Discussion

SABATINO is the first survey conducted in Romania specifically investigating the adult patients’ expectations and attitudes towards asthma and its treatment. It shows that despite advances in asthma therapy, significant unmet needs persist in terms of asthma care, particularly in those with severe disease and points towards a lack of improvement in asthma control.

Asthma symptoms and exacerbations were common in the sample analysed; for almost half of the patients, physicians reported ≥2 exacerbations and for a quarter of them≥2 hospitalizations within 12 months prior to the survey. Moreover, one-third of the patients reported ER visits. The situation was more dramatic when data were analysed according to the asthma severity, with severe asthma patients having more exacerbations and hospitalizations than mild-to-moderate ones. Unsurprisingly, severe asthma patients used significantly more reliever medication (including over-the-counter use) than mild-to-moderate patients. These results align with previously published results of surveys performed in patients with asthma, which also showed a persistence of significant exacerbations and low levels of symptom control in other European populations [11,12]. It is known that patients with severe asthma are a category characterized by a high burden of illness due to poor symptom control, experiencing frequent and often life-threatening exacerbations, associated comorbidities, and low quality of life [20,21,22]. A recent study evaluated the experiences and impact of severe asthma on patient’s life and showed significant emotional distress in these patients because of the disease and its therapy [21]. This study identified the neglected needs of patients with severe asthma, such as “empathy and understanding” and “encouragement” (21). It also pointed towards the need for a support service that would improve adherence problems resulting in concerns about medication side effects [21].

The behaviour of SABATINO participants who reported the use of reliever therapy only to prevent exacerbation or of maintenance therapy when experiencing symptoms is not uncommon [13,14,15]. Previous reports suggested that low adherence to the prescribed therapy probably reflects patients’ beliefs about medication and their personality traits [13,23,24]. In our survey, we identified 3 attitude clusters corresponding to different personality traits with distinct clinical characteristics. “Empowered savvy” had the lowest frequency of severe asthma, the highest adherence to maintenance therapy and the highest level of confidence in the effectiveness of asthma medication. The opposite of this attitude cluster is the “anxious strugglers” with more patients with severe asthma, a higher score for worries about asthma therapy (side effects, dose, and appropriateness especially when symptoms were absent) and better knowledge of their treatment as self-reported which was in contradiction with their behaviour, with 25% of them reporting taking maintenance therapy only when having breathing difficulties. The clusters identified in SABATINO show similarities to clusters previously identified in other populations, which reported well-controlled asthma among patients with few concerns about their medications [25,26]. The non-confidence in the effectiveness of asthma medication and negative concerns about therapy were associated with reduced adherence to therapy [16] and uncontrolled disease [26]. These findings suggest that asthma management should not only be tailored for the severity of the disease but should also consider patients’ beliefs and behaviours, specifically targeting medication concerns with the aim to improve treatment adherence [24,27].

The lack of adherence to prescribed therapy and empowerment in asthma self-management may also reflect the discrepancy between treatment goals as seen by patients and physicians and between different asthma severity groups, suggesting different patients’ needs. For example, the most important treatment goal reported by the highest percentage of physicians was preventing asthma exacerbations, while for patients it was the participation in activities of daily living. The difference in asthma expectations between patients and physicians when it comes to asthma control is not new, and points toward unmet patient needs [28,29,30]. Previous studies showed that physicians tend to focus on asthma control while patients are more concerned about long-term health and costs [28,29,30]. When analysed by the severity of the disease, in our survey, the most important goals identified by the highest percentage of patients was participation in daily-life activities for those with mild and moderate asthma and preventing chronic symptoms that interfere with daily lives in those with severe asthma. These results indicate a different valuation of treatment goals that vary according to the severity of the disease and the need for targeted approaches. The one-size-fit-all approach may not be suitable, and physicians should work with their respiratory patients to define individualized treatment goals through a shared-decision making process.

This survey has several limitations that may limit the generalization of our findings. This was a cross-sectional survey, and the selection bias cannot be precluded. Moreover, the sample size, especially of those with severe asthma was limited. It was not designed to compare patients with different asthma severity, but it would be of interest to observe these differences in future surveys designed for this purpose.

## 5. Conclusions

The results of this survey point to suboptimal asthma control in Romania and underlines the significant burden of asthma, and especially of severe asthma, in Romania, with implications for clinical practice and policymakers. The different valuations of the treatment goals observed in patients and physicians, and in different asthma severity groups suggest the need for individualized approaches, more patient-centred. Guidelines recommendations should be adapted, with practical tools more adequate for the Romanian healthcare setting to be provided for the routine use of clinicians, including patient educational programs. The ultimate common goal should be to improve patients’ knowledge and self-awareness through a solid therapeutic alliance with the treating physicians, thus enabling optimal symptom control.

## Figures and Tables

**Table 1:** Respondents’ demographic and clinical characteristics.

**Patients Characteristics**	***N* = 433**
Women, *n* (%)	247 (57.0%)
Age, *n* (%)	
>55 years	160 (37.0%)
40–55 years	164 (37.9%)
<40 years	109 (25.2%)
Smokers, *n* (%)	102 (23.6%)
Education, *n* (%)	
University education	154 (35.6%)
High school	214 (49.4%)
Primary or secondary school	63 (14.5%)
Occupational status, *n* (%)	
Employed	261 (60.3%)
Retired	129 (29.8%)
Unemployed	21 (4.8%)
Pupil/student	13 (3.0%)
Housewife	8 (1.8%)
Paid leave/maternity leave	1 (0.2%)
**Physicians characteristics**	***N* = 70**
Specialty, *n* (%)	
Pulmonology	55 (78.6%)
Allergology	15 (21.4%)
Years of experience, *n* (%)	
<15 years	29 (41.4%)
15–20 years	22 (31.4%)
>20 years	19 (27.1%)
Region of Romania, *n* (%)	
Bucharest	25 (35.7%)
East	14 (20.0%)
West	9 (12.8%)
South	12 (17.1%)
Center	10 (14.3%)
Average number of asthma patients/month, *n* (%)	
>50 patients	24 (34.3%)
30–50 patients	28 (40.0%)
<30 patients	18 (25.7%)

*n*/*N* (%), number (percentage) of patients.

**Table 2:** Clinical characteristics of patients and indicators of asthma symptoms and exacerbations according to asthma severity.

	Mild *N* = 84	Moderate *N* = 262	Severe *N* = 87	Total *N* = 433	*p*-Value for Difference between Groups
**Physician Reported Characteristics**					
Years since asthma diagnosis, mean ± SD	6.9 ± 6.0	8.1 ± 7.8	10.9 ± 9.2	8.5 ± 7.9	0.002
Number of exacerbations during the past 12 months, mean ± SD	1.0 ± 1.0	1.5 ± 1.3	3.2 ± 1.9	1.8 ± 1.5	<0.001
Distribution of number of exacerbations during the past 12 months, *n* (%)					<0.001
No exacerbation	28 (33.3%)	50 (19.1%)	2 (2.3%)	80 (18.5%)	
1 exacerbation	34 (40.5%)	85 (32.4%)	12 (14.0%)	131 (30.3%)	
2 exacerbations	11 (13.1%)	79 (30.2%)	16 (18.0%)	106 (24.5%)	
≥3 exacerbations	6 (7.1%)	42 (16.0%)	56 (64.0%)	104 (24.0%)	
Number of hospitalizations during the past 12 months, mean ± SD	0.6 ± 1.5	0.8 ± 1.2	2.0 ± 1.8	1.0 ± 1.5	<0.001
Distribution of number of hospitalizations in the past 12 months, *n* (%)					<0.001
No hospitalization	51 (60.7%)	127 (48.5%)	14 (16.1%)	192 (44.3%)	
1 hospitalization	17 (20.2%)	70 (26.7%)	23 (26.4%)	110 (25.4%)	
2 hospitalizations	4 (4.8%)	42 (16.0%)	26 (29.9%)	72 (16.6%)	
≥3 hospitalizations	5 (6.0%)	13 (5.0%)	23 (26.4%)	41 (9.5%)	
**Patients reported characteristics**					
Patients with ER visits during the past 12 months, *n* (%)	17 (20.2%)	66 (25.2%)	57 (65.5%)	140 (32.3%)	<0.001
Patients with overnight hospital stay during the past 12 months, *n* (%)	26 (31.0%)	104 (39.7%)	62 (71.3%)	192 (44.3%)	<0.001
No of days with inability to work or carry out usual activities during the past 12 months, *n* (%)					<0.001
0–2 days	42 (50.0%)	114 (43.5%)	9 (10.3%)	165 (38.1%)	
3–5 days	17 (20.2%)	53 (20.2%)	15 (17.2%)	85 (19.6%)	
6–9 days	8 (9.5%)	17 (6.5%)	6 (6.9%)	31 (7.2%)	
≥10 days	3 (3.6%)	14 (5.3%)	27 (31.0%)	44 (10.2%)	
Do not remember	14 (16.7%)	64 (24.4%)	30 (34.5%)	108 (24.9%)	
Asthma impact on patient’s activities during last month, *n* (%)					<0.001
High & Very high	3 (3.6%)	17 (6.5%)	15 (17.2%)	35 (8.1%)	
Moderate	10 (11.9%)	68 (26.0%)	29 (33.3%)	107 (24.7%)	
Limited	24 (28.6%)	83 (31.7%)	32 (36.8%)	139 (32.1%)	
Very limited	21 (25.0%)	60 (22.9%)	10 (11.5%)	91 (21.0%)	
None	26 (31.0%)	34 (13.0%)	1 (1.1%)	61 (14.1%)	

ER, emergency room; *n*/*N* (%), number (percentage) of patients; SD, standard deviation.

**Table 3:** Asthma therapy usage as reported by patients and physicians.

	Mild *N* = 84	Moderate *N* = 262	Severe *N* = 87	Total *N* = 433	*p*-Value for Difference between Groups
**Physician Reported Characteristics**					
Maintenance therapy type, *n* (%)					
ICS/LABA ^#^	48 (57.1%)	200 (76.3%)	72 (82.8%)	320 (73.9%)	
ICS	23 (27.4%)	28 (10.7%)	20 (23.0%)	71 (16.4%)	
Antileukotrienes	14 (16.7%)	66 (25.2%)	39 (44.8%)	119 (27.5%)	
Anticholinergic	3 (3.6%)	10 (3.8%)	20 (23.0%)	33 (7.6%)	
Xanthines	2 (2.4%)	10 (3.8%)	4 (4.6%)	16 (3.7%)	
OCS	1 (1.2%)	15 (5.7%)	24 (27.6%)	40 (9.2%)	
Biological therapy	0 (0.0%)	2 (0.8%)	8 (9.2%)	10 (2.3%)	
Reliever therapy (SABA), *n* (%)	60 (71.4%)	215 (82.1%)	80 (92.0%)	355 (82.0%)	
**Patients reported characteristics**					
Mean number of reliever inhalers purchased in the previous 12 months, mean ± SD	3.1 ± 2.6	3.1 ± 2.2	5.1 ± 3.4	3.6 ± 2.8	<0.001
Mean number of maintenance inhalers purchased in the previous 12 months, mean ± SD	8.0 ± 4.2	8.4 ± 4.0	7.7 ± 4.3	8.2 ± 4.1	0.51
Usage of reliever inhalers during last month, *n* (%)					<0.001
Never	46 (54.8%)	86 (32.8%)	17 (19.5%)	149 (34.4%)	
Some weeks	26 (31.0%)	148 (56.5%)	31 (35.6%)	205 (47.3%)	
Every week	12 (14.3%)	28 (10.7%)	39 (44.8%)	79 (18.2%)	
Over-the-counter reliever therapy, *n* (%)	28 (33.3%)	79 (30.2%)	42 (48.3%)	149 (34.4%)	0.008

# Only as combination therapy. GP, general practitioner; ICS, inhaled corticosteroids; LABA, long-acting beta agonists; *n*/*N* (%), number (percentage) of patients; OCS, oral corticosteroids; SABA, short-acting beta agonists; SD, standard deviation

**Table 4:** Patient characteristics for each attitude cluster identified.

	Empowered Savvy *N* = 158	Pessimistic Non-Compliers *N* = 187	Anxious Strugglers *N* = 88	*p*-Value for Difference between Groups
Age, years	49.0 ± 13.4	52.0 ± 13.9	46.0 ± 15.7	0.002
Smokers, *n* (%)	28 (17.7%)	39 (20.9%)	35 (39.8%)	<0.001
Education, *n* (%)				0.003
University education	75 (47.5%)	49 (26.2%)	30 (34.1%)	
Highschool	62 (39.2%)	110 (58.8%)	42 (47.7%)	
Primary or secondary school	21 (13.3%)	27 (14.4%)	15 (17.0%)	
Asthma severity, *n* (%)				0.003
Mild	42 (26.6%)	28 (15.0%)	14 (15.9%)	
Moderate	94 (59.5%)	121 (64.7%)	47 (53.4%)	
Severe	22 (13.9%)	38 (20.3%)	27 (30.7%)	
Number of exacerbations during the past 12 months, mean ± SD	1.5 ± 1.7	1.7 ± 1.4	2.4 ± 1.4	<0.001
Number of hospitalizations during the past 12 months, mean ± SD	0.6 ± 1.7	1.1 ± 1.1	1.7 ± 1.4	<0.001
Maintenance therapy type, *n* (%)				
ICS/LABA *	132 (83.5%)	127 (67.9%)	61 (69.3%)	
Antihistamines	24 (15.2%)	61 (32.6%)	42 (47.7%)	
Antileukotrienes	44 (27.8%)	51 (27.3%)	24 (27.3%)	
ICS	14 (8.9%)	23 (12.3%)	34 (38.6%)	
OCS	12 (7.6%)	11 (5.9%)	17 (19.3%)	
Reliever therapy (SABA), *n* (%)	123 (77.8%)	165 (88.2%)	67 (76.1%)	<0.05
Usage of asthma therapy, *n* (%)				<0.001
Always	146 (92.4%)	147 (78.6%)	80 (90.9%)	
Sometimes	12 (7.6%)	40 (21.4%)	8 (9.1%)	
Reliever therapy use, *n* (%)				0.002
When coughing or having breathing difficulties	139 (88.0%)	145 (77.5%)	61 (69.3%)	
Sometimes to prevent asthma exacerbations	18 (11.4%)	38 (20.3%)	27 (30.7%)	
Usage of reliever inhalers during last month, *n* (%)	91 (57.6%)	125 (66.8%)	68 (77.3%)	0.007
Mean number of reliever inhalers purchased in the previous 12 months, mean ± SD	3.4 ± 2.5	3.6 ± 2.9	3.9 ± 2.8	0.448
Maintenance therapy use, *n* (%)				0.001
Every day	140 (88.6%)	140 (74.9%)	51 (58.0%)	
When coughing or having breathing difficulties	15 (9.5%)	36 (19.3%)	22 (25.0%)	
Mean number of maintenance inhalers purchased in the previous 12 months, mean ± SD	10.1 ± 3.3	7.3 ± 4.0	5.5 ± 4.0	<0.001

* Only as combination therapy. ICS, inhaled corticosteroids; LABA, long-acting beta agonists; *n*/*N* (%), number (percentage) of patients; OCS, oral corticosteroids; SABA, short-acting beta agonists; SD, standard deviation.

## Data Availability

Research data are not shared.

## Supplementary Materials

The following are available online at https://www.mdpi.com/article/10.3390/medicina57101089/s1, Supplementary Table S1. Questionnaires used for physicians and patients in the SABATINO survey, Supplementary Table S2. Patients’ attitudes toward asthma by severity of asthma, Supplementary Table S3. Patients’ attitude toward asthma according to attitude clusters identified
