# Supplementary material for: Patient and Physician Perspectives on Asthma and Its Therapy in Romania: Results of a Multicenter Survey
Source: Medicina (Kaunas). 2021 Oct 12;57(10):1089. doi: 10.3390/medicina57101089 (PMC8541282; doi:10.3390/medicina57101089)
Supplement: Supplementary file 1 [file medicina-57-01089-s001.zip › medicina-1392703-supplementary.pdf]

Supplementary Material

Table S1. Questionnaires used for physicians and patients in the SABATINO survey.

Questionnaire used for physicians

Q0. Which is your primary medical specialty? *[single answer]*

1	Pulmonology	**CONTINUE**
2	Allergology	
3	Other medical specialty	**STOP INTERVIEW**

Q1. For how long have you been practicing this medical specialty? *[write the number]*

	.... years	***TERMINATE if Q1<3 or Q1>35***

Q2. How many patients diagnosed with asthma do you see on average in a regular month? *[write the number]*

	.... patients	**STOP INTERVIEW if Q2<20**

Q3. Here are some treatment goals that specialists may take into consideration when treating patients diagnosed with bronchial asthma. Please rank these treatment goals according to the importance for you, where 5 means "the most important treatment goal" and 1 means "the least important treatment goal". *[answers from 1 to 5] [randomize]*

1	Allowing the patient to participate in all activities of daily living (work, school, exercise, etc)	I__I
2	Preventing asthma attacks/ exacerbations	I__I
3	Providing the best medicine treatment for patients	I__I
4	Avoiding adverse effects from asthma medications	I__I
5	Preventing long-term (chronic) symptoms that interfere with activities of daily living, such as coughing or shortness of breath in the morning, during daytime or after exercise	I__I
6	Decreasing nighttime symptoms and achieve uninterrupted sleep	I__I
7	Maintaining pulmonary function as close to normal levels as possible	I__I
8	Preventing asthma mortality	I__I

Questions for physicians regarding each patient enrolled

Q1. Patient was diagnosed with … asthma. *[single answer]*

1	Mild
2	Moderate
3	Severe
4	Very severe

Q2. Patient is now suffering from … asthma. *[single answer]*

1	Mild
2	Moderate
3	Severe
4	Very severe
**Q3. Does the patient have any allergies?** *[single answer]*	
1	Yes
2	No
**Q4.** *[Ask if Q3=1]* **What type of allergies does this patient have?** *[single answer]*	
1	Pollen allergy
2	House dust allergy
3	Drug allergy
4	Food allergy
5	Insect/ cockroach allergy
6	Pet allergy
7	Other types of allergies, please specify: ....
**Q5. Number of exacerbations in the past 12 months.** *[single answer]*	
1	.... exacerbations
2	I am not fully aware of this information
**Q6. Number of hospitalizations in the past 12 months.** *[single answer]*	
1	.... hospitalizations
2	I am not fully aware of this information
**Q7. Current treatment followed by this patient.** *[multiple answer]*	
A	ICS
B	ICS/LABA

C	SABA	Salbutamol	1
		Ventolin	2
		Berotec	3
		Other	4
D	Anticholinergic		

F	Antileukotrienes
G	OCS
H	Xanthines
I	Antihistamines
J	Biological therapy

ICS, inhaled corticosteroids; LABA, long-acting beta agonists; OCS, oral corticosteroids; SABA, short-acting beta agonists

Questionnaire used for patients		
**Q1. Please fill-in the following information about yourself.** *[write your answers on each row]*		
1	Your initials.	....
2	Age.	.... years old
3	Gender.<br>*Single answer.*	1. Male<br>2. Female
5	What is the last form of education that you graduated from?<br>*Single answer.*	1. Primary/secondary<br>2. Medium<br>3. High<br>4. Don't know/ No answer
6	What is your occupation and position?<br>*Single answer.*	1. I have a permanent job<br>2. I have a temporary job<br>3. Unemployed<br>4. Retired<br>5. Pupil/ student<br>6. Housewife<br>7. Maternity leave/ paid leave<br>5. Do not work from other reasons
7	Do you smoke?<br>*Single answer.*	1. Yes<br>8. No
ASTHMA DIAGNOSIS		
**Q2. How many years ago have you been diagnosed with asthma?** *[write the number]*		
	.... years	
**Q3. Which of the following physicians diagnosed you with asthma?** *[single answer]*		
1	Pulmonologist	
2	Allergist	
3	General practitioner	
4	Internal medicine physician	

5	Other medical specialty, please specify: ....
**Q4. You have received your asthma diagnosis as a result of …** *[multiple answer] [randomize]*	
1	Cough symptoms
2	Wheezing symptoms
3	Chest tightness
4	Breathlessness
5	Fatigue
6	Asthma attack
7	Other, please specify: ....
**Q5. Do you usually cough or have hard time breathing after being around asthma triggers such as?** *[multiple answer] [randomize]*	
1	Smoke
2	Animals / pets
3	House dust
4	Cockroaches
5	Grass / flowers
6	Molds
7	Chalk dust
8	Strong smells / perfume
9	Foods
10	Having a cold
11	Stress or emotional upsets
12	Changes in temperature
13	No, I haven't coughed or had hard time breathing after being around asthma triggers
**Q6. What do you do when you have breathing problems?** *[multiple answer] [randomize]*	
1	Stop and rest
2	Drink something
3	Do deep slow breathing
4	Take my quick-relief inhaler (meaning the treatment taken only when needed when my symptoms worsen)
5	Increase my maintenance medication (meaning the day-by-day treatment recommended by my doctor for asthma control)

6	Call my doctor or nurse
7	Go to the emergency room or hospital
8	Other, please specify: ....

ASTHMA TREATMENT HABITS		
**Q7. Do you take any medication for your asthma?** *[multiple answer]*		
1	Yes	*CONTINUE INTERVIEW*
2	Sometimes	
3	No	*STOP INTERVIEW*

**Q8. Your treatment plan includes...** *[single answer]*	
1	Only maintenance therapy (meaning the day-by-day treatment recommended by my doctor for asthma control)
2	Only reliever therapy (meaning the treatment taken only when needed when my symptoms worsen)
3	Both

**Q9. [***Complete if you checked Q8=1 or Q8=3]* **How clear is to you the way in which you should use your maintenance therapy?** *[single answer]*	
1	Not clear at all
2	Rather unclear
3	Neither clear, nor unclear
4	Rather clear
5	Very clear

**Q10.** *[Complete if you checked Q8=2 or Q8=3]* **How clear is to you the way in which you should use your reliever therapy?** *[single answer]*	
1	Not clear at all
2	Rather unclear
3	Neither clear, nor unclear
4	Rather clear
5	Very clear

**Q11.** *[Complete if you checked Q8=1 or Q8=3]* **How clear is to you the role/ mode of action of your maintenance therapy?** *[single answer]*	
1	Not clear at all
2	Rather unclear
3	Neither clear, nor unclear

4	Rather clear
5	Very clear

Q12. *[Complete if you checked Q8=2 or Q8=3]* **How clear is to you the role/ mode of action of your reliever therapy?** *[single answer]*

1	Not clear at all
2	Rather unclear
3	Neither clear, nor unclear
4	Rather clear
5	Very clear

Q13. *[Complete if you checked Q8=1 or Q8=3]* **When do you usually take the maintenance medication for your asthma?** *[single answer]*

1	Only when I cough or have breathing problems
2	Every day, even when I am feeling well, to prevent asthma symptoms
3	Other, please specify: ....

Q14. *[Complete if you checked Q8=2 or Q8=3]* **When do you usually take the reliever medication for your asthma?** *[single answer]*

1	Only when I cough or have breathing problems
2	Sometimes, even when I am feeling well, to prevent asthma symptoms
3	Other, please specify: ....

Q15. *[Complete if you checked Q8=2 or Q8=3]* **When you feel that you are having asthma-related breathing problems, what reliever medication do you use?** *[multiple answers possible]*

Reliever medication	Salbutamol	1
	Ventolin	2
	Berotec	3
	Something else/ I don't remember	4

Q16. *[Complete if you checked Q8=2 or Q8=3]* **How many items/ devices of <u>reliever therapy</u> have you bought or picked up on receipt during the past 12 months?** *[write the number]*

............items/ devices bought in the past 12 months

Q17. *[Complete if you checked Q8=1 or Q8=3]* **How many items/ devices of <u>maintenance therapy</u> have you bought or picked up on receipt during the past 12 months?** *[write the number]*

............Items/ devices bought in the past 12 months

Q18. Were there situations in which your GP recommended (issued you a prescription) for...?

1	Maintenance therapy
2	Reliever therapy

3	Both

Q19. Were there situations in which you bought your reliever medication directly from the pharmacy (without a prior recommendation from a physician)?

1	Yes
2	No

Q20. Here are some aspects that patients suffering from asthma may expect from the treatment they take. Please rank these expectations/aspects according to the importance they have for you, where 5 means "the most important aspect" and 1 means "the least important aspect". *[answers from 1 to 5] [randomize]*

1	Allowing to participate in all activities of daily living, including work, school, and exercise	I__I
2	Preventing asthma attacks/ exacerbations	I__I
3	Having access to the best medicine treatment for asthma	I__I
4	Avoid adverse effects from asthma medications	I__I
5	Preventing long-term (chronic) symptoms that interfere with daily living, such as coughing or shortness of breath in the morning, during daytime or after exercise	I__I
6	Decrease nighttime symptoms and achieve uninterrupted sleep	I__I
7	Maintaining pulmonary function as close to normal levels as possible	I__I

ASTHMA ATTACKS/ EXACERBATIONS

Q21. Today, how would you evaluate your current health status when it comes to your asthma symptoms/ asthma control? *[single answer]*

1<br>Very poor	2<br>Poor	3<br>Relatively poor	4<br>Neither poor, nor good	5<br>Relatively good	6<br>Good	7<br>Very good

Q22. Have you used your reliever medication during the past month? *[single answer]*

1	Yes, I have used it every week in the past month
2	Yes, I have used it in some weeks and in others I haven't
3	No, I haven't used my reliever medication in the past month

Q23. *[Complete if you checked Q21=1]* **How many times have you used your reliever medication in the past month?** *[write the number]*

…….times

Q24. In general, during the past month, how limited were you in your activities because of your asthma? *[single answer]*

7	Not at all
6	Very slightly
5	Slightly

4	Moderately
3	Very
2	Extremely
1	Totally

Q25. During the past year, how many times have your asthma caused you to …? *[multiple answer]*

1	Stay overnight in the hospital	.... times
2	Go to the emergency room	.... times
3	Go to the medical clinic to see a specialist	.... times
4	Go to your general practitioner	.... times
5	Have periods of symptoms' worsening but which did not require to visit the doctor or to be hospitalized	.... times

Q26. During the past year, how many days were you unable to work or carry out your usual activities because of your asthma symptoms? *[single answer]*

1	0 days
2	1-2 days
3	3-5 days
4	6-9 days
5	10-14 days
6	15 days or more

ATTITUDES TOWARDS ASTHMA

Q27. Please indicate how strongly you agree or disagree with the following statements, using a scale from 1 to 7, where 1 means "Strongly disagree" and 7 means "Strongly agree". *[single answer on each row]*

		Strongly disagree						Strongly agree	*DN/NA*
	**EFFECTIVENESS**								
1	I feel in control of my disease	1	2	3	4	5	6	7	*99*
2	When I take my medication, I feel confident that my asthma symptoms will be controlled	1	2	3	4	5	6	7	*99*
3	I feel happy to continue my current asthma medication	1	2	3	4	5	6	7	*99*
	**EASE OF USE**								
4	Following of my asthma medication is easy	1	2	3	4	5	6	7	*99*

5	I rarely leave home without taking my reliever inhaler(s) with me	1	2	3	4	5	6	7	*99*
6	It is easy to remember to take my maintenance medication for asthma every day	1	2	3	4	5	6	7	*99*
	**SIDE-EFFECTS AND WORRIES**								
7	I worry that my inhaler is not giving me enough medication	1	2	3	4	5	6	7	*99*
8	I worry that I am not taking the right medication for my symptoms	1	2	3	4	5	6	7	*99*
9	I have concerns about the potential side effects of my asthma medication	1	2	3	4	5	6	7	*99*
	**BURDEN OF ASTHMA MEDICA-TION**								
10	I wish my asthma medication was eas-ier to take	1	2	3	4	5	6	7	*99*
11	Using more than one inhaler can be a hassle or a problem	1	2	3	4	5	6	7	*99*
12	Using my asthma inhaler(s) in public is embarrassing	1	2	3	4	5	6	7	*99*
13	My asthma medication is too expen-sive for me	1	2	3	4	5	6	7	*99*
	**ASTHMA CONTROL & TREAT-MENT HABITS**								
14	I know my asthma well enough to in-tervene early and prevent a worsening of symptoms or an asthma attack	1	2	3	4	5	6	7	*99*
15	I can manage by myself a worsening of symptoms or an asthma attack without going to the doctor	1	2	3	4	5	6	7	*99*
16	I prefer to adjust my asthma medica-tion, taking less when feeling well and more when feeling worse	1	2	3	4	5	6	7	*99*
17	I am concerned that I am taking too much medication when I feel well or I have no asthma symptoms	1	2	3	4	5	6	7	*99*
18	I feel scared / worried about having breathing problems	1	2	3	4	5	6	7	*99*

SOURCES OF INFORMATION

Q28. Which are your sources of information for information about asthma? *[multiple answer]*
Q29. *[Complete for sources checked at Q28]* **Which sources of information do you trust the most? Please rank the first 5 sources of information you trust the most using a scale from 1 to 5, where 1 means "the 1st most trusted source of information", 2 means "the 2nd most trusted source of information", 3 means "the 3rd most trusted source of information" and so on.** *[order answers from 1 to 5]*

1	General practitioner	1	|______|
2	Specialist physician	2	|______|
3	Nurse	3	|______|
4	Pharmacist	4	|______|
5	Relatives	5	|______|
6	Friends	6	|______|
7	Internet search engines (Google, Yahoo etc.)	7	|______|
8	Specific disease or health websites	8	|______|
10	Online heath forums	10	|______|
11	Pharmaceutical companies' websites	11	|______|
12	Facebook	12	|______|
13	Instagram	13	|______|
14	YouTube	14	|______|
15	TV	15	|______|
16	Radio	16	|______|
17	Magazines	17	|______|
18	Other source of information, please specify:	18	|______|
99	None, I don't look for asthma information	99	

Table S2. Patients' attitudes toward asthma by severity of asthma

	Mild N=84	Moderate N=262	Severe N=87	Total N=433
**Effectiveness**				
I am willing to continue the administration of my asthma medication	6.2±1.2	6.0±1.4	6.4±1.0	6.1±1.3
When I take medication, I feel confident that symptoms will be controlled	5.9±1.4	5.6±1.5	5.9±1.2	5.7±1.5
I feel in control of my disease	5.4±1.7	5.2±1.8	4.9±1.7	5.2±1.7
**Ease of use**				
It is easy to remember to take my maintenance medication every day	5.7±1.6	5.5±1.6	5.6±1.6	5.6±1.6
Keeping track of my asthma medication is easy	5.7±1.4	5.4±1.6	5.4±1.4	5.4±1.5
I rarely leave home without taking my	5.3±1.8	5.2±1.8	5.6±1.7	5.3±1.8

reliever inhaler(s) with me				
**Concerns about therapy**				
I worry about the potential side effects of my asthma medication	3.1±1.9	3.4±2.1	4.3±2.1	3.5±2.1
I worry that my inhaler doesn't give me enough medication	2.9±1.9	3.4±2.0	4.3±2.2	3.5±2.1
I worry about not taking the right medication for my symptoms	2.6±1.8	3.1±2.1	4.1±2.2	3.2±2.1
**Burden of asthma medication**				
I wish my asthma medication were easier to take	4.3±2.1	4.9±1.9	5.5±1.8	4.9±1.9
My asthma medication is too expensive for me	4.1±2.1	4.4±1.9	4.9±1.9	4.5±1.9
Using more than one inhaler may be a hassle or a problem	3.9±2.2	4.3±1.9	4.9±1.9	4.3±2.0
Using my asthma inhaler(s) in public is embarrassing	3.4±2.1	3.7±2.0	4.2±2.2	3.7±2.1
**Control and treatment habits**				
I know my asthma well enough to prevent a worsening of symptoms	5.1±2.0	5.1±1.7	5.2±1.3	5.1±1.7
I am scared and/ or worried about having breathing problems	4.6±2.1	4.8±1.9	5.5±1.7	4.9±1.9
I can manage by myself a worsening of symptoms without going to doctor	4.6±2.0	4.6±1.7	4.7±1.7	4.6±1.7
I am worried about taking too much medication when I feel well	3.2±2.1	3.6±2.1	4.7±1.9	3.8±2.1
I prefer to adjust my asthma medication	3.4±2.3	3.6±2.2	4.3±2.1	3.7±2.2

All data in this table are presented as mean ± SD. N, number of patients.

Table S3. Patients' attitude toward asthma according to attitude clusters identified

	Empowered Savvy N=158	Pessimistic Non-Compliers N=187	Anxious Strugglers N=88
**Effectiveness**			
I am willing to continue the administration of my asthma medication	6.7±0.7	5.5±1.7	6.0±1.5
When I take medication, I feel confident that symptoms will be controlled	6.5±0.7	4.9±1.6	5.8±1.7
I feel in control of my disease	6.2±1.2	4.0±1.7	5.4±1.8
**Ease of use**			
It is easy to remember to take my maintenance medication every day	6.3±1.2	4.8±1.6	5.5±1.7
Keeping track of my asthma medication is easy	6.4±0.8	4.6±1.5	5.3±1.7

I rarely leave home without taking my reliever inhaler(s) with me	5.9±1.7	4.6±1.7	5.4±1.9
**Concerns about therapy**			
I worry about the potential side effects of my asthma medication	2.0±1.4	4.0±1.9	5.2±1.8
I worry that my inhaler doesn't give me enough medication	1.6±1.0	4.0±1.8	5.3±1.8
I worry about not taking the right medication for my symptoms	1.4±0.8	3.7±1.7	5.3±1.9
**Burden of asthma medication**			
I wish my asthma medication were easier to take	3.9±2.1	5.2±1.7	5.7±1.7
My asthma medication is too expensive for me	3.6±2.0	5.3±1.6	3.9±2.0
Using more than one inhaler may be a hassle or a problem	3.1±2.0	5.0±1.7	4.3±2.3
Using my asthma inhaler(s) in public is embarrassing	3.1±2.0	4.8±1.9	2.3±1.6
**Control and treatment habits**			
I know my asthma well enough to prevent a worsening of symptoms	5.9±1.6	4.3±1.7	5.0±1.7
I am scared and/ or worried about having breathing problems	3.9±2.3	5.5±1.3	5.2±2.0
I can manage by myself a worsening of symptoms without going to doctor	4.9±1.9	4.2±1.7	4.7±1.9
I am worried about taking too much medication when I feel well	2.5±1.9	4.0±1.9	5.6±1.5
I prefer to adjust my asthma medication	2.9±2.1	3.5±2.0	5.4±1.8
